# Cloning of Toll-like Receptor 3 Gene from *Schizothorax prenanti* (*SpTLR3*), and Expressions of Seven *SpTLRs* and *SpMyD88* after Lipopolysaccharide Induction

**DOI:** 10.3390/genes13101862

**Published:** 2022-10-15

**Authors:** Jiqin Huang, Jianlu Zhang, Kunyang Zhang, Cheng Fang, Wanchun Li, Qijun Wang

**Affiliations:** 1 Shaanxi Key Laboratory of Qinling Ecological Security, Shaanxi Institute of Zoology, Xi’an 710032, China; 2 Key Laboratory for Hydrobiology in Liaoning Province, College of Fisheries and Life Science, Dalian Ocean University, Dalian 116023, China; 3 Hanzhong Nanzheng District Aquatic Product Workstation, Hanzhong 723100, China

**Keywords:** clone, gene expression, toll-like receptor 3, lipopolysaccharide, *Schizothorax prenanti*

## Abstract

Toll-like receptor 3 (*SpTLR3*) from *Schizothorax prenanti* (*S. prenanti*) was cloned and identified, and the tissue distribution of the *SpTLR3* gene was examined in this study. Moreover, the relative mRNA expression levels of myeloid differentiation factor 88 gene (*SpMyD88*) and seven TLR genes (*SpTLR2*, *SpTLR3*, *SpTLR4*, *SpTLR18*, *SpTLR22-1*, *SpTLR22-2* and *SpTLR22-3*) from *S. prenanti* after lipopolysaccharide (LPS) challenge were analyzed through quantitative real-time polymerase chain reaction (qRT-PCR). The full length of *SpTLR3* gene is 3097 bp, and complete coding sequence (CDS) is 2715 bp, which encodes 904 amino acids. The *Sp*TLR3 amino acid sequence shared 43.94–100% identity with TLR3 sequences from other vertebrates; *SpTLR3* was expressed in all eight tissues examined; and the highest level appeared in the liver, which was significantly higher than in all other tissues (*p* < 0.05), followed by the levels in the heart and muscles. LPS significantly up-regulated all eight genes in the *S. prenanti* tissues at 12 or 24 h (*p* < 0.05). Compared with the PBS control group, no significant transcripts changes were found in *SpTLR2* or *SpTLR3* at 12 h after LPS induction, but they were significantly up-regulated at 24 h (*p* < 0.001). The most abundant transcripts were found in the head kidney *SpTLR22* genes after 24 h LPS induction, with high to low levels, which were *SpTLR22-1* (564-fold), *SpTLR22-3* (508-fold) and *SpTLR22-2* (351-fold). Among these eight genes, the expression level of *SpTLR4* was the least up-regulated. Overall, *SpTLR4* in the head kidney was involved in the antibacterial immune response earlier, and the level was increased at 12 h with extreme significance after LPS stimulation (*p* < 0.001), while the other seven genes were the most significantly up-regulated at 24 h post injection. Taken together, the results suggest that *SpMyD88*, *SpTLR2*, *SpTLR3*, *SpTLR4*, *SpTLR18*, *SpTLR22-1*, *SpTLR22-2* and *SpTLR22-3* participate in an innate immune response stimulated by LPS, and the response intensity of the genes was organ-specific, with differing kinetics. Our findings will contribute to a more complete understanding of the roles of these TLR genes in antibacterial immunity.

## 1. Introduction

The vertebrate immune system includes the innate immune system and the adaptive immune system. In mammals, the adaptive immune system plays a major role in the eventual elimination of pathogens. However, the difference is that fish belong to poikilotherm, and body temperature is not suitable for the development of acquired immune function. Therefore, the innate immune system plays a “leading role” in fish immunity [1]. Similar to mammals, the innate immunity of fish is mainly due to a series of evolutionarily highly conserved pattern recognition receptors (PRRs). The PRRs recognize pathogen-associated molecular patterns (PAMPs), which are conserved on the surface of microorganisms but not present in the host body. The invasion signal is transmitted to the whole host immune system, and the immune signal is transmitted to the cell, thus inducing a disease-resistant immune response [2,3]. Toll-like receptors (TLRs) were first identified in *Drosophila melanogaster* by Anderson et al. in 1985 [4]. Subsequently, human TLR4 was the first toll-receptor discovered in mammals and was thought to be homologous to TLR1 in *D. melanogaster* [5,6]. During the same period, TLRs have been gradually discovered in different vertebrates, and the first IL-1R/TLR superfamily member in fish was discovered by Sangrador-Vegas et al. in 2000 [7]. To date, 13 TLRs have been identified in mammals and classified into six TLR subfamilies, namely, TLR1, TLR3, TLR4, TLR5, TLR7 and TLR11, and at least 22 TLRs have been cloned and identified in teleost fish (TLR1-4, 5M, 5S, 7-9, 13-14, and 18-28), which also belong to the six TLR subfamilies [8], some of which are teleost-specific TLRs, such as TLR18-28. As the main PRRs, these TLRs play an important role in identifying microbial pathogens that infect fish. Together with interferon regulatory factors (IRFs), myeloid differentiation factor 88 (MyD88) and other factors in the immune signaling pathway, TLRs participate in the recognition process of bacteria, viruses, parasites and most other pathogenic microorganisms [9,10,11,12]. The fish species are complex, and teleostei experience one whole genome duplication, which greatly increases the diversity of genotypes and the complexity of gene functions [13]. The TLR system for sensing microorganisms has similarities with mammal, and also important and diverse in teleostei, especially for teleost-specific TLRs [12]. The study of fish TLRs is of great significance for understanding the immune system of lower vertebrates.

During TLR signal transduction, adapter molecules are recruited by the Toll/IL-1 receptor (TIR) domain of TLR, leading to the signal entry into different pathways. The TLR signaling pathways can be broadly divided into two categories, including MyD88-dependent and MyD88-independent pathways. In the MyD88-dependent pathway, as an important adaptor protein, MyD88 is recruited by TLRs as the first signal protein and plays a highly important role in TLR signal transduction [14,15,16].

In mammals, TLR3 receives dsRNA stimulatory signals to mediate antiviral immune responses, and fish TLR3 has a similar function [17]. Studies showed that Poly (I:C) up-regulated the expression of TLR3 gene in rainbow trout (*Oncorhynchus mykiss*) [18] and golden pompano (*Trachinotus ovatus*) [19], respectively, especially in immune organs. Su et al. [20] reported that TLR3 transcription levels in spleen and skin of grass carp (*Ctenopharyngodon idella*) were significantly increased after Grass Carp Reovirus (GCRV) infection. Notably, the fish TLR3 subfamily has only one member, TLR3. Similar to mammalian, fish TLR3 mainly recognizes viral nucleic acids, but it has also been shown to be involved in anti-bacterial immunity [17,21]. In addition, *Sp*TLR2 and *Sp*TLR18 involved in this study belong to the TLR1 subfamily, *Sp*TLR4 belongs to the TLR4 subfamily, and *Sp*TLR22s belongs to the TLR11 subfamily [8]. Lipopolysaccharide (LPS) is a major component of the outer membranes of Gram-negative bacteria, which can induce a cascade of immune stimulation and toxic pathophysiological activities in the body [22]. Whether LPS induces changes in *S.* *prenanti* TLR3 expression is unknown. Therefore, the *SpTLR3* was cloned for the first time in this study, and the specific domains of the TLR3 family were analyzed. The secondary structure composition and the 3D structural models of *Sp*TLR3 protein were predicted. Moreover, the expression patterns of different immune organs in response to the LPS stimulation of the seven *SpTLRs* and *SpMyD88* gene were analyzed using qRT- PCR.

*S.prenanti* is a unique cold-water fish in China, which belongs to Cyprinidae, the subfamily of schizothorinae, and the genus *Schizothorax*, and is a rare and high-quality fish in producing areas. In Hanzhong, Shaanxi province, *S.* *prenanti* is known locally as “Yang-fish”, whereas in Ya’an, Sichuan province, *S.* *prenanti* and the other special economic fish *S. davidi* are collectively known as “Ya fish” [23,24,25]. Due to intensive feeding, *S. prenanti* is susceptible to bacterial infection, such as *Aeromonas hydrophila* [26] and *Streptococcus agalactiae* [27], which seriously impedes the healthy development of the Ya-fish aquaculture. To date, three *SpTLR22* molecules (named *SpTLR22-1*, *SpTLR22-2* and *SpTLR22-3*) have been cloned and identified [14], as well as two *SpTLR5* family members (named *SpTLR5-1* and *SpTLR5-2*) [28] and *SpTLR25* [29]. We treated *S. prenanti* with LPS intraperitoneal injection in this study, and examined the relative mRNA expression of *MyD88*, *TLR2*, *TLR3*, *TLR4*, *TLR18*, *TLR22-1*, *TLR22-2* and *TLR22-3* genes from *S. prenanti* in different immune organs (hepatopantras, head kidney, hindgut and spleen) at different infection times, which will help to further clarify the roles of *SpTLRs* and *SpMyD88* genes involved in the antibacterial immunity mechanisms of fish.

## 2. Materials and Methods

### 2.1. Animal Treatment

We obtained healthy cultured *S. prenanti* (109.3 ± 27.1 g) in November 2020 from a professional breeding cooperative (Qunfu Yang-Fish, Hanzhong, China), which is a Chinese national aquatic germplasm resources breeding farm and commercial breeding aqua-farm. The fish were maintained in glass tanks with a size of 60 × 40 × 30 cm^3^ and with aerated water at a temperature of 19 ± 1 °C; The tank filter was cleaned and about a quarter of the aerated tap water was replaced daily. The fish were fed with a commercial feed (Floating compound feed with crude protein ≥ 32%, crude fat ≥ 3%) at a rate of 2% of their body weight twice daily.

The fish were anesthetized with eugenol at a concentration of 80 mg/L for 3 min before dissected. The heart, liver, head kidney, hindgut, muscle, intraperitoneal fat, spleen and gill were collected from eugenol anaesthetized fish to detect the tissue distribution of *SpTLR3* mRNA in unstressed conditions. To understand the expression of *SpMyD88*, *SpTLR2*, *SpTLR3*, *SpTLR4*, *SpTLR18*, and *SpTLR22*s (*SpTLR22-1*, *SpTLR22-2*, *SpTLR22-3*) in response to LPS stimulation, the fish were challenged with an intraperitoneal injection of LPS (10 mg/kg), and the control group was injected with phosphate-buffered saline (PBS) at the same volume. The liver, head kidney, hindgut and spleen were collected from four *S.prenanti* of PBS–injection and LPS treatment at 12 and 24 h, respectively. The dissected tissues and preserved in liquid nitrogen for RNA extraction.

### 2.2. RNA Extraction and cDNA Synthesis

Tissue total RNA was extracted using the TaKaRa MiniBEST Universal RNA Extraction Kit (TaKaRa, Dalian, China). The total RNA concentration and purity were determined by RNA electrophoresis and the optical density absorption ratio (A260/280) in the Nanodrop One spectrophotometer (Thermo Fisher Scientific, Wilmington, NC, USA). First-stand cDNA for *SpTLR3* partial sequence amplification was synthesized using the RevertAid First Strand cDNA Synthesis Kit (Thermo Scientific, Vilnius, Lithuania), according to the method recommended by the manufacturer. The *SpTLR3* 5′-rapid amplification of cDNA ends (RACE) cDNA was prepared using the SMARTer^®^ RACE 5′/3′ kit (TaKaRa, Dalian, China).

### 2.3. Full Length Cloning of the SpTLR3

According to the transcriptome sequencing of *S. prenanti*, the specific *SpTLR3* partial sequence primers were designed (Table 1, TLR3-F and TLR3-R) and synthetized by Tsingke Biotechnology Co., Ltd. (Xi’an, China). Spleen cDNA was used as a template for *SpTLR3* amplification, with Primerstar^®^ Max DNA polymerase (TaKaRa, Dalian, China). The PCR program was as follows: 35 cycles at 98 °C for 10 s, 55 °C for 15 s, and 72 °C for 40 s. The PCR products were ligated into a lineareized pRACE vector (TaKaRa, Dalian, China), and transformed into Stellar Competent Cells (TaKaRa, Dalian, China). The positive bacteria clones were sequenced. According to the obtained *SpTLR3* partial sequence, the primer for 5′-RACE was designed (Table 1, GSP) and synthetized by Tsingke Biotechnology Co., Ltd. (Xi’an, China). Spleen cDNA was used as a template for *SpTLR3* amplification, with SeqAmp DNA polymerase (TaKaRa, Dalian, China). The touchdown PCR program was as follows: 5 cylces at 94 °C for 30 s, 72 °C for 3 min; 5 cycles at 94 °C for 30 s, 70 °C for 30 s, and 72 °C for 3 min; 25 cycles at 94 °C for 30 s, 68 °C for 30 s, and 72 °C for 3 min. The PCR products were ligated into a lineareized pRACE vector (TaKaRa, Dalian, China), and transformed into Stellar Competent Cells (TaKaRa, Dalian, China). The positive bacteria clones were sequenced.

### 2.4. Sequence Analysis

The *SpTLR3* complete CDS was identified using the ORF Finder (http://www.ncbi.nlm.nih.gov/gorf/gorf.html, accessed on 2 September 2022). The isoelectric point and molecular weight were predicted (https://web.expasy.org/compute_pi/, accessed on 2 September 2022). The signal peptide, Leucine-rich repeats (LRRs), transmembrane domain and TIR domain of *Sp*TLR3 was predicted by the SMART programs (http://smart.embl-heidelberg.de/, accessed on 7 September 2022). The secondary structure composition of *Sp*TLR3 protein was predicted (https://www.novopro.cn/tools/secondary-structure-prediction.html, accessed on 2 September 2022). The three-dimensional structure of *Sp*TLR3 protein was predicted (https://swissmodel.expasy.org/, accessed on 2 September 2022). Multiple sequence alignments were performed using the Clustal X2.1 [30]. A phylogenetic tree of different vertebrates TLR3 based on amino acid sequences was constructed by the neighbor-joining method using MEGA 4.0 software [31].

### 2.5. Tissue Distribution of TLR3 mRNA in Unstressed Conditions

The measured tissues in control group included heart, liver, spleen, head kidney, muscle, intraperitoneal fat, hindgut and gill. Total RNA was extracted and cDNA was synthesized, as described previously (“RNA extraction and cDNA synthesis”). The qRT-PCR analysis was performed using the applied Biosystems Step One Plus (Life Technologies, Foster City, CA, USA). The gene-specific primers are listed in Table 1. *S. prenanti*-specific β-actin primers were used to normalize the cDNA quantity for each tissue sample. Quantification of *SpTLR3* and β-actin was performed in triplicate on all samples using FastStart Essential DNA Green Master (Roche Diagnostics, Risch-Rotkreuz, Switzerland), according to the manufacturer’s instructions. The qRT-PCR data were calculated according to the 2^−ΔΔCT^ method [32].

### 2.6. Detection of the Expression Patterns Induced by LPS

For the LPS challenge, 8 fish were injected intraperitoneally from the base of the pectoral fin with LPS (Sigma, L2880). The dose of injection was 10 mg/kg LPS per fish. Another 8 fish were injected intraperitoneally PBS at the same amount, as a control. 4 fish samples were taken at 12 and 24 h after LPS injection respectively. In generally, head kidney, liver, and hindgut of fish are regarded as immune organs and central to the immune responses [33], the three tissues plus spleen, another vital immune organ were collected from each fish and used to isolate total RNA. To detect the expression of *SpMyD88*, *SpTLR2*, *SpTLR 3*, *SpTLR4*, *SpTLR18*, *SpTLR22-1*, *SpTLR22-2* and *SpTLR22-3* changes after LPS induction, total RNA extraction, cDNA synthesis, and qRT-PCR were performed, as described previously (Tissue distribution of *SpTLR3* mRNA in unstressed conditions). *S. prenanti*-specific β-actin primers were used to normalize the cDNA quantity for each tissue sample. The quantitative primers of *SpTLR2*, *SpTLR4*, *SpMyD88* were designed according to the transcriptome sequencing of *S. prenanti*. The quantitative primers of *SpTLR22-1*, *SpTLR22-2* and *SpTLR22-3* were designed according to the gene sequences unloaded to GenBank (GenBank accesion no. MN082612, MN082613, MN082614). Quantitative primers of *SpTLR18* refer to the article published by Li et al. [34]. The quantitative primers used are listed in Table 1.

### 2.7. Statistical Analysis

SPSS 22.0 and Graphpad Prism 5.0 software was used for data analysis and histogram, respectively. The mRNA expression levels were analyzed by using One-way ANOVA method. All data are presented as the mean ± standard error (n = 4), and the statistically significant differences between PBS control and LPS treatment groups at each time point are expressed with asterisks, * *p* < 0.05, ** *p* < 0.01, *** *p* < 0.001 vs. corresponding control group at the time points.

## 3. Results

### 3.1. Identification, Structural, and Phylogenetic Analysis of SpTLR3

**Figure 1:**
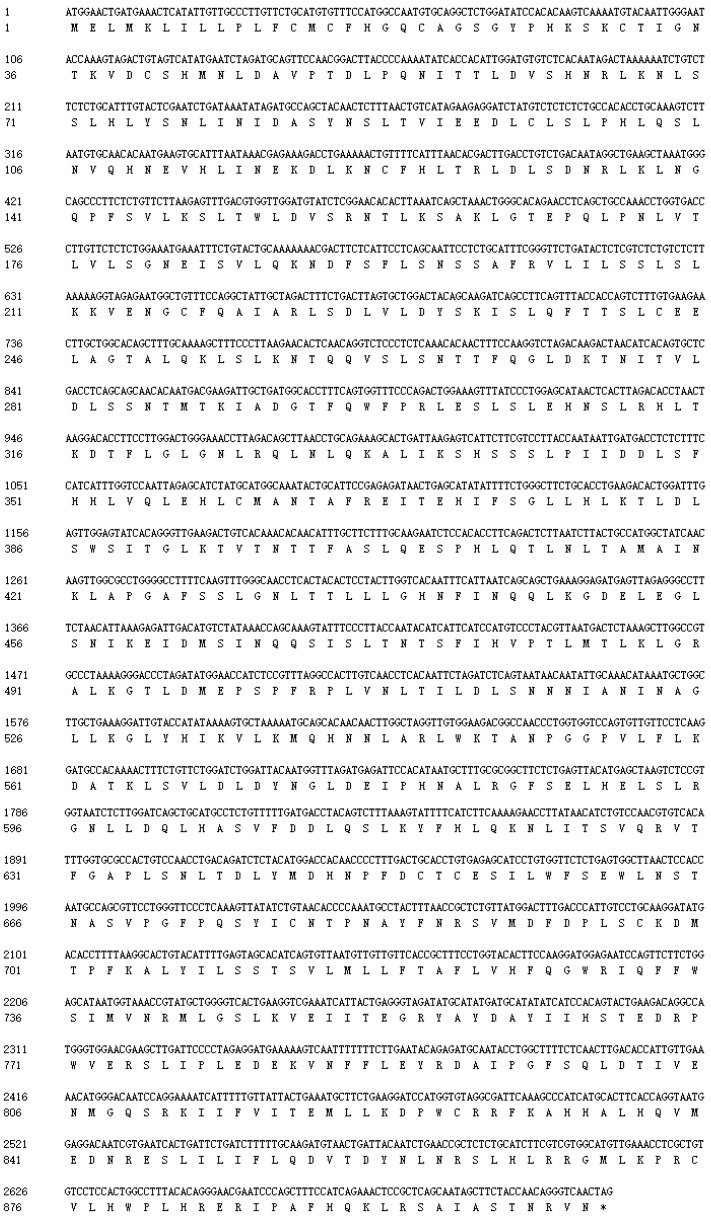
Nucleotide and deduced amino acid sequences of *Sp*TLR3. * Stop codon.

**Figure 2:**
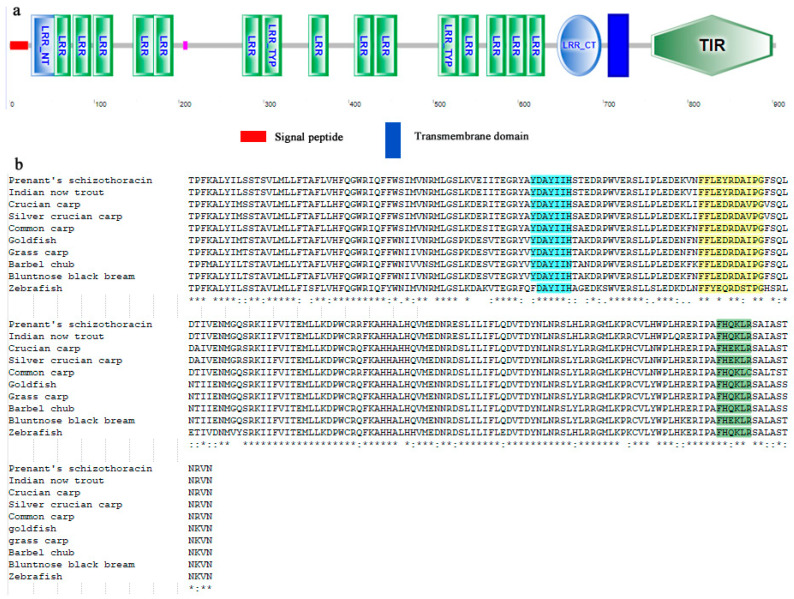
The predicted domain of *Sp*TLR3 protein by SMART programs. (**a**) Schematic representation of TLR3 domains predicted; (**b**) Multiple alignment of TLR3-TIR domains from different species. Snow trout (GenBank accession no. AGJ74274), Crucian carp (GenBank accession no. AGO57935), Silver crucian carp (GenBank accession no. AGR53439), Carp (GenBank accession no. AHE74142), Goldfish (GenBank accession no. ABC86865), Grass carp (GenBank accession no. ABI64155), Barbel chub (GenBank accession no.ALO75529), Bluntnose black bream (GenBank accession no. ABI83673) and Zebrafish (GenBank accession no. AAI07956). Asterisk (*), identical; colon (:), conserved; and dot (.), semi-conserved residues. The conserved box1 is shown in blue, the conserved box2 is shown in yellow and the conserved box3 is shown in green.

**Figure 3:**
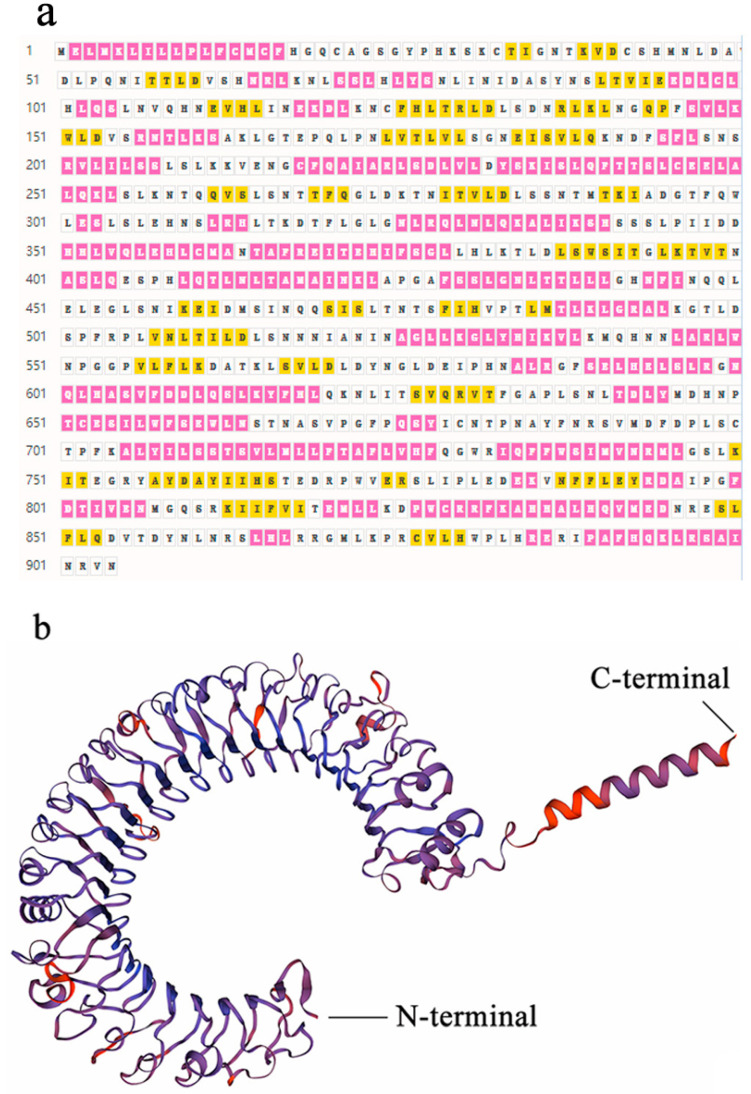
Predicted secondary structure and 3D structural models of *Sp*TLR3 protein. (**a**) Predicted secondary structure of *Sp*TLR3 protein. ■ indicates helix, ☐ indicates coil, ■ indicates strand. (**b**) Three-dimensional model structures of predicted *Sp*TLR3 protein.

**Figure 4:**
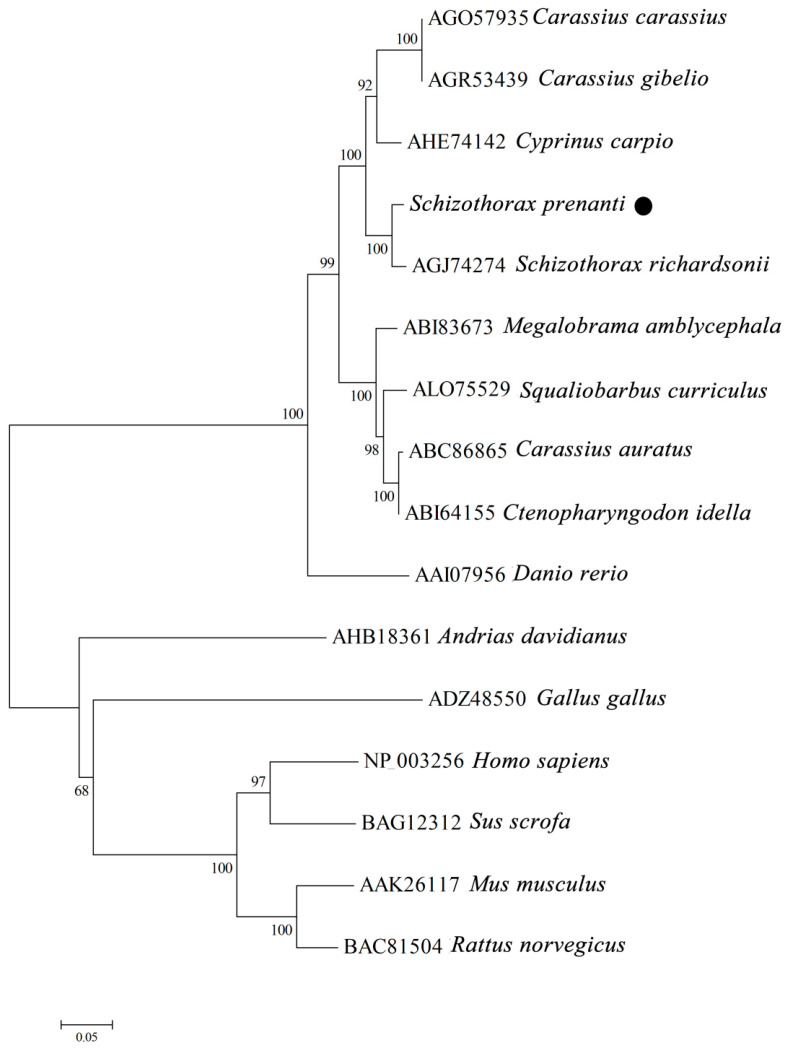
Phylogenetic tree of relationships between *S. prenanti* TLR3 and other vertebrates. Tree was constructed by neighbor-joining method using MEGA 4.0 software. Numbers at nodes indicate proportions of bootstrapping after 1,000 replications. ● *S. prenanti* TLR3.

The full length of *SpTLR3* gene is 3097 bp (GenBank accesion no. OP589305), including 312 bp of 5’ untranslated region (UTR), 2715 bp of complete CDS (Figure 1) and 70 bp 3’ UTR. The predicted open reading frame (ORF) encoded a protein of 904 amino acids, including one signal peptide, seventeen LRRs, one transmembrane domain and one TIR domain (Figure 2a). The calculated molecular mass and theoretical isoelectric point of the *Sp*TLR3 is 102.29 kDa and 7.08, respectively. Like the TIR domain of other fish TLR3, *Sp*TLR3 has three conserved regions: Box 1, Box 2 and Box 3 (Figure 2b), indicating that the TIR domain is relatively conserved throughout TLR3 in fish. Figure 3a shows the distribution of strands, helixes, and coils, and Figure 3b shows the predicted 3D structure of the *Sp*TLR3 protein. The *Sp*TLR3 amino acid sequence was the most similar to those of other fish and was the closest to snow trout (*Schizothorax richardsonii*) TLR3, with 100% identity. We analyzed the phylogeny of the *Sp*TLR3 amino acid sequences to determine the evolutionary relationships between *Sp*TLR3 and TLR3 from other vertebrates, based on sequences in the GenBank database (Figure 4). The results revealed a high amino acid sequence identity between *Sp*TLR3 and the TLR3 of *Cyprinids*. Moreover, *Sp*TLR3 and the TLR3 of *S. richardsonii* have the same amino acid sequence identity as the two fish belong to the same genus *Schizothorax* (Teleostei: Cyprinidae).

### 3.2. Tissue Distribution of SpTLR3 Expression in S. prenanti

**Figure 5:**
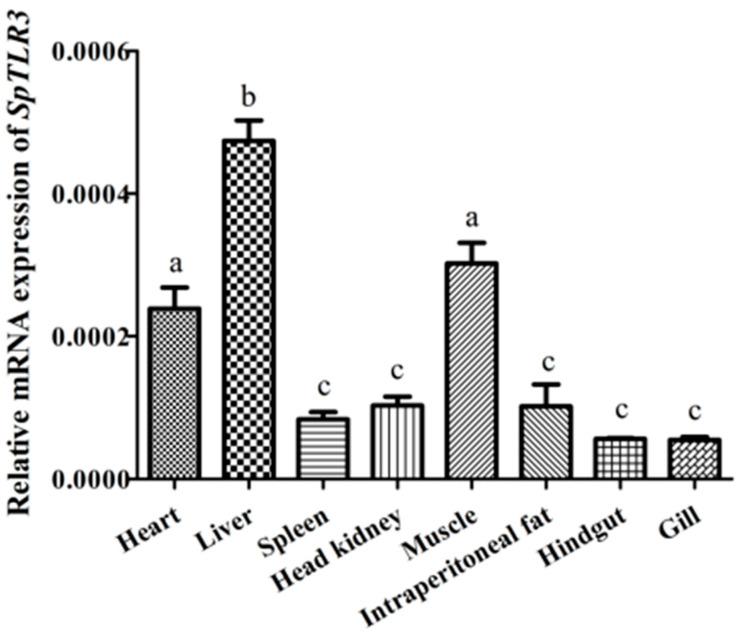
Abundance of *SpTLR3* transcripts in heart, liver, spleen, head kidney, muscle, intraperitoneal fat, and intestine of *S. prenanti* was determined by qRT-PCR. The loading control for normalization was β-actin. The a, b, c means with different letters are significantly different from each other (*p* < 0.05). Values are shown as the means ± standard error (n = 4). Error bars, standard error of the means (n = 4 fish per group).

We quantified *SpTLR3* mRNA expression in the heart, liver, spleen, head kidney, muscle, intraperitoneal fat, hindgut and gill tissue from four fish using qRT-PCR to determine *SpTLR3* transcript expression. The loading control for normalization was β-actin. Figure 5 shows the ubiquitous, but variable expression of *SpTLR3* transcripts in all eight tissues. The highest expression level of *SpTLR3* appeared in the liver, and the level was significantly higher than in all other tissues (*p* < 0.05). Furthermore, the expression of *SpTLR3* in the heart and muscle was more pronounced than that in the spleen, head kidney, intraperitoneal fat, hindgut and gill (*p* < 0.05). The expression *SpTLR3* did not significantly differ in the spleen, head kidney, intraperitoneal fat, hindgut and gill tissues.

### 3.3. Expression of SpMyD88 and SpTLRs after Injection of LPS

To determine the change in *MyD88*, *TLR2*, *TLR3*, *TLR4*, *TLR18*, *TLR22-1*, *TLR22-2* and *TLR22-3* from *S. prenanti* after LPS infection at 12 and 24 h, the mRNA levels of the genes in the liver, head kidney, spleen and hindgut were quantified using qRT-PCR.

#### 3.3.1. Expression of SpMyD88 after Injection of LPS

**Figure 6:**
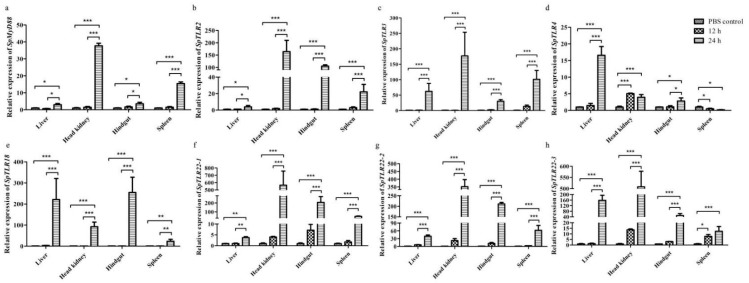
The relative expression levels of *SpMyD88* (**a**), *SpTLR2* (**b**), *SpTLR3* (**c**), *SpTLR4* (**d**), *SpTLR18* (**e**), *SpTLR22-1* (**f**), *SpTLR22-2* (**g**) and *SpTLR22-3* (**h**) transcripts in liver, head kidney, hindgut, and spleen of *S. prenanti* at 12 and 24 h after LPS injection determined by qRT-PCR. Values were normalized using β-actin. Data are expressed as the means ± standard error (n = 4). Statistically significant differences between the groups are indicated by asterisks (* *p* < 0.05, ** *p* < 0.01, *** *p* < 0.001). PBS, phosphate-buffered saline.

The expression of SpMyD88 in the four tissues examined showed no significant change after the treatment of S. prenanti with LPS 12 h later (Figure 6a). However, at 24 h of LPS infection, the gene was significantly up-regulated in all four tissues, especially in the head kidney and spleen tissues, which showed an exponentially up-regulated trend compared with the PBS control group and the 12 h infection group (*p* < 0.001). Moreover, in the liver and hindgut, SpMyD88 of the 24 h infection group was significantly higher than that in the PBS control group and 12 h infection group (*p* < 0.01).

#### 3.3.2. Expression of SpTLR2 after Injection with LPS

At 12 h after LPS infection, SpTLR2 expression in all four tissues examined showed no significant change, which was similar to the expression pattern of SpMyD88. However, at 24 h after LPS challenge, the expression level of SpTLR2 in the liver was significantly increased (*p* < 0.05), whereas the other three tissues were extremely significant up-regulated (*p* < 0.001). In particular, the expression level of the gene was extremely high in the head kidney, followed by the hindgut and the liver, showing that the expression levels of the SpTLR2 gene in the tissues after infection were extremely different (Figure 6b).

#### 3.3.3. Expression Levels of SpTLR3 after Injection with LPS

After LPS treatment, the expression pattern of SpTLR3 in both the 12 h infection group and the 24 h infection group was similar to the pattern of SpTLR2; that is, compared with the PBS control group, the expression level of SpTLR3 in the LPS 12 h infection group showed no significant change in the four tissues, but the level in the 24 h infection group was significantly higher than that in the PBS and 12 h infection group (*p* < 0.001), and the highest expression level was found in the head kidney, followed by the spleen and hindgut (Figure 6c).

#### 3.3.4. Expression of SpTLR4 after Injection with LPS

After LPS infection, SpTLR4 expression was different from that of the above genes. Compared with the PBS group, the SpTLR4 of the liver and hindgut in the 12 h infection group showed no significant change, while that of the head kidney tissue was significantly up-regulated (*p* < 0.001), and the high expression level persisted until 24 h after infection. The levels of the 24 h infection group were as follows: the gene expression of SpTLR4 in the liver was exponentially up-regulated and more strongly significant than that of the PBS group and 12 h infection group (*p* < 0.001). The level in the head kidney remained high in the 24 h infection group, which was significantly higher than that in the PBS group (*p* < 0.001). The level in the hindgut was significantly higher than that in the PBS control group and 12 h infection group (*p* < 0.05). The difference is that the expression level in the spleen was downregulated, with a highly significant difference in both the 12 h and 24 h infection groups compared to in the PBS control group (*p* < 0.05) (Figure 6d).

#### 3.3.5. Expression of SpTLR18 after Injection with LPS

The temporal expression pattern of SpTLR18 was similar to that of SpTLR2, SpTLR3 and SpMyD88; that is, no significant difference was found between the 12 h infection group and the PBS group. The SpTLR18 gene was overexpressed in the liver, head kidney and hindgut, with significant levels of *p* < 0.001. The level of SpTLR18 in the spleen was relatively low, but showed a significance level of *p* < 0.01 (Figure 6e).

#### 3.3.6. Expression of SpTLR22s after Injection with LPS

As shown in Figure 6f–h, the temporal expression patterns of *SpTLR22* genes (*SpTLR22-1*, *SpTLR22-2* and *SpTLR22-3*) in the four organs examined after LPS stimulation were consistent. After 12 h, in the fish challenged with LPS, no significant changes were found compared with the PBS-injection group; however, the levels of *SpTLR22-3* in the spleen were significantly up-regulated (*p* < 0.05). In contrast, the highest transcripts were found in the liver post-24 h LPS injection for all three *SpTLR22s*, followed by the levels of *SpTLR22-1* and *SpTLR22-2* in the hindgut, spleen and liver, and then by the levels of *SpTLR22-3* in the liver, hindgut and spleen. Furthermore, the expression levels of all *SpTLR22s* in the four organs spiked after 24 h LPS stimulation, and the level of *SpTLR22-1* in liver was significantly higher than that in the PBS control and 12 h groups (*p* < 0.01), whereas the levels of other *SpTLR22s* in different organs were much higher than those in the PBS and 12 h groups (*p* < 0.001).

## 4. Discussion

In this study, we cloned the *SpTLR3* gene for the first time, The predicted *Sp*TLR3 amino acid sequence included the conserved typical structure of TLR protein family: 17 LRR domain, Transmembrane domain and TIR domain. The previous studies have demonstrated that the LRR domain was related to recognizing the pathogen components and the number of LRR domain differs in different animals [17,35] For instance, 16 LRR domains were found in Japanese flounder [17]. In the present study, there were three conserved regions: Box 1, Box 2 and Box 3, indicating that the TIR domain is relatively conserved throughout TLR3 in fish. Box1 and Box2 play an important role in mediating receptor binding to downstream signaling molecules, while Box3 directly controls the expression and localization of receptor molecules [36]. Multiple sequence alignment showed that the *Sp*TLR3 protein is moderately conserved, and that its amino acid sequence is highly similar to that of *Cyprinids*. The results of the *Sp*TLR3 amino acid sequencing and phylogenetic tree analysis revealed that *S. prenanti* is more closely related to *S. richardsonii*. This was consistent with previous findings of *Sp*TLR5-1 [28], whereas *Sp*TLR5-2 [28] and *Sp*TLR22s (*Sp*TLR22-1, *Sp*TLR22-2, and *Sp*TLR22-3) [37] were more closely related to common carp (*Cyprinus carpio*). Additionally, *S. prenanti* apelin receptor (APJ) [38] and protein nucleobindin-2A (NUCB2A) [39] were more closely related to zebrafish (*Danio rerio*) and goldfish (*Carassius auratus*).

MyD88 is an important transmitter of the TLR signaling pathway, which is highly conserved and plays an indispensable role in both TLR signaling pathway and (Interleukin-1) IL-1 receptor family. It goes through a series of signal transductions after the MyD88-depended pathway is activated by TLRs recognition, that lead to transcription factors such as Nuclear factor-κB (NF-κB), activator protein-1 (AP-1) and interferon regulatory factors (IRFs), eventually triggering inflammatory responses [14,40]. To date, few studies on fish MyD88 have been conducted. Takano et al. [41] found that the number of MyD88-positive cells in the kidney and spleen increased three days after Japanese flounder (*Paralichthys olivaceus*) was infected with *Edwardsiella tarda*. The yellow drum (*Nibea albiflora*) was treated with *Pseudomonas plecoglossicida*, then, the *NaMyD88* in the head kidney was rapidly up-regulated and remained significantly higher than that in the PBS control group from 2 to 24 h after infection, indicating that the gene plays an important role in the innate immunity of the fish [42]. These results are similar to that of *SpMyD88* in our study. Moreover, when the yellow croaker (*Pseudosciaena crocea*) was infected with *Vibrio parahaemolyticus*, the expression level of spleen *PcMyD88* was significantly higher at 24 and 48 h than that of the control group, and the highest expression level appeared at 48 h post-injection [43]. It can be concluded that MyD88 plays a crucial role in the innate immunity of fishes, whereas the time of immune response varies with different pathogens in different species.

To date, a total of 13 TLRs (TLR1-13) have been identified in mammals and classified into 6 TLR subfamilies, named TLR1, TLR3, TLR4, TLR5, TLR7 and TLR11 subfamilies [44], and there are 22 TLRs (TLR1-4, 5M, 5S, 7-9, 13-14, and 18-28) in teleost fish have been identified, which also belong to the 6 TLR subfamilies [8]. The classification of the 6 fish TLR subfamilies are as follows: TLR1 (TLR1, 2, 14, 18, 24, 25, 27 and 28), TLR3 (TLR3), TLR4 (TLR4), TLR5 (TLR5M and TLR5S), TLR7 (TLR7, 8 and 9), and TLR11 (TLR13, 19, 20, 21, 22, 23 and 26) subfamily [8,45,46]. In mammals, TLR2 typically functions as a dimer, recognizing different components of bacteria [45]. Like mammals, the TLR2 of channel catfish (*Ictalurus punctatus*) can also act as a dimer with TLR1 [47], and the TLR2 of orange-spotted grouper (*Epinephelus coioides*) participates in the anti-LPS or Poly(I:C) immune response [48]. Our study confirmed that the *SpTLR2* participates in antibacterial immunity, and the best monitoring organ is the head kidney. TLR18 is a fish-specific TLR. To date, the TLR18 of zebrafish (*D. rerio*) [49], channel catfish (*I. punctatus*) [45], Atlantic salmon (*Salmo salar*) [50], grass carp [51], yellow catfish (*Pelteobagrus fulvidraco*) [52] and common carp (*Cyprinus carpio L*.) [53] have been identified. These reports and the present study have together proved that the *TLR18* gene plays an important role in the innate immune responses of teleost fish. However, the *TLR18* expression levels in different tissues vary from fish to fish. In this study, the highest expression abundance of *SpTLR18* after LPS stimulation was found in the liver.

TLR3 is the only member of the fish TLR3 subfamily [17], mainly involved in mediating antiviral immunity [18,19,20]. However, fish TLR3 can also senses bacterial stimulation. It was reported that *TLR3* mRNA levels in the liver, kidney and spleen of channel catfish were significantly up-regulated at different time points after infection with *Edwardsiella ictaluri* [21]. However, there was no significant change in the *TLR3* of zebrafish infected with *Mycobacterium marinum* [48], although the expression of *TLR3* was significantly down-regulated in Rainbow trout infected with *Yersinia reuteri* [54]. In the current study, the expression level of *SpTLR3* increased exponentially in the head kidney, spleen and hindgut after 24 h LPS injection. In conclusion, the response of fish TLR3 to PAMPs may depend on many factors, such as time, host species and pathogens.

Similar to TLR3, TLR4 is also the only member of the TLR4 subfamily of fish, and in mammals, TLR4 is the direct receptor of bacterial LPS. The biggest difference between TLR signaling pathway in fish and that in mammals lies in the TLR4-mediated signaling pathway [54]. Moreover, TLR4 is only present in a few fish [49]. Zebrafish TLR4 has been demonstrated not only to fail to recognize LPS challenge in vitro, but also have no responsive to heat-killed *Escherichia coli* and *Legionella pneumophila* [55,56]. Moreover, TLR4 negatively regulated NF-κB activation; therefore, the LPS recognition system in fish is quite different from that in mammals [46,56]. In contrast, it was reported that both LPS and *A. hydrophila* (a Gram-negative fish pathogen) infection could significantly up-regulate the expression of TLR4 from rohu (*Labeo rohita*) [57]. Our study confirmed the following three conclusions. First, the TLR4 in *S. prenanti* is present. Second, the *Sp*TLR4 is involved in anti-LPS immunity, which is consistent with the conclusion of Samanta et al. [57], but different from that of Sullivan et al. [55] and Sepulcre et al. [56]. Finally, the liver is the most sensitive organ for *SpTLR4* detection. The expression level of *SpTLR4* was the least up-regulated among the eight genes examined, which was in fact the most different *Sp*TLRs in our study. It was the only one that up-regulated at 12 h in head kidney. Those most different results verified the specificity of fish TLR4, and the immune interpretation need further study.

In general, the expression levels of the *SpTLR22s* were the highest in the seven induced *SpTLRs* and *SpMyD88* examined in the present study. TLR22, which was also one of the TLRs specific to fish, belonging to the TLR11 subfamily, first discovered in goldfish in 2003, The TLR22 of goldfish macrophages was highly up-regulated by LPS, heat-killed *Aeromonas salmonicida*, and live *Mycobacterium chelonei* [58]. Subsequently, TLR22 has been cloned and reported in nearly 20 fish species, such as zebrafish [49], Japanese flounder [59], Atlantic salmon [60], yellowtail (*Seriola lalandi*) [61], and common carp [62]. To date, most of the studies reporting on TLR22 function have focused on bacteria, viruses, parasite infection and PAMP stimulation. It has been found that TLR22 is a multi-functional immune receptor that participates in almost all the defensive immune responses of pathogenic microorganisms. The results of *SpTLR22s* in this study also confirmed these conclusions. Moreover, two subtypes of TLR22 (namely, *TLR22-1* and *TLR22-2*) were first discovered in Rainbow trout in 2007, which have very similar functions and were called ‘twin’ TLRs, and the expression of the two *OmTLR22s* in peripheral leukocytes was significantly up-regulated after stimulation by *A. salmonicida* [63]. Subsequently, three copies of the *TLR22* gene (namely, *TLR22-1*, *TLR22-2* and *TLR22-3*) were found in Atlantic salmon (GenBank accession numbers: AM233509, FM206383 and BT045774), and even up to twelve *TLR22* gene copies (namely, *TLR22a~l*) have been found in Atlantic cod (*Gadus morhua*) [64]. Du et al. reported that both *SpTLR22-1* and *SpTLR22-3* in the head kidney and spleen respond to the stimulation of LPS and *A. hydrophila*, but not *SpTLR22-2*, in the two immune tissues [14]; this was different from our findings, as we found that the *SpTLR22-2* levels in the four organs were significantly up-regulated at 24 h after LPS stimulation. Moreover, *SpTLR22-1* in the head kidney was significantly up-regulated at 12 h after LPS challenge, but no pronounced significance was found at the 24 h time point [14], showing a different temporal expression pattern to our findings, in addition to the expression in the spleen. Notably, the temporal expressions of *SpTLR22s* in our study showed similar kinetics to one another in different immune organs, indicating that the functions of the subtypes of *SpTLR22*s are highly similar. Our results may indicate that both the *SpTLR22*s jointly mediate the recognition of LPS and participate in the immune response.

## 5. Conclusions

In this study, *SpTLR3* was cloned and identified for the first time, and the secondary structure composition, the 3D structural models of the *Sp*TLR3 protein, as well as the binding site were predicted. Furthermore, the *SpTLR3* gene was expressed in all the tissues examined and mainly expressed in immune-related tissues. Phylogenetic analysis showed that the *Sp*TLR3 protein is most closely related to TLR3 from snow trout. Multiple sequence alignment showed that the *Sp*TLR3 is moderately conserved. Among the seven immune tissues we examined, the distribution of *SpTLR3* was the most abundant in the liver, followed by the kidney and spleen. Moreover, the LPS organ specifically induced *SpTLR2*, *SpTLR3*, *SpTLR4*, *SpTLR18*, *SpTLR22s* (*SpTLR22-1*, *SpTLR22-2*, and *SpTLR22-3*) and *SpMyD88* at 12 or 24 h, which are involved in antibacterial immunity. It is worth noting that the *SpTLR22*s were the most sensitive to LPS induction.

## Figures and Tables

**Table 1:** The primers used for *SpTLR3* clone and qRT-PCR.

Primer	Sequence (5′-3′)	Annealing Temperature (°C)	Size (bp)
Primers for partial sequence cloning			
TLR3-F	CAGACTCTTAATCTTACTG	55	1558
TLR3-R	GACATACCAAATAAGGAC		
Primers for 5′RACE			
GSP	CGACCTTCAGTGACCCCAGCATACGG	Touchdown PCR	2543
UPM (kit provided)	CTAATACGACTCACTATAGGGCAAG CAGTGGTATCAACGCAGAGT		
Primers for qRT-PCR			
TLR2-F	GATCAACGGCACAGTGTTTG	62	170
TLR2-R	CAGGTCTGAAAGGAGGTTCTG		
TLR3-F	GCTGAAAGGAGATGAGTTAGAG	62	110
TLR3-R	ACGTAGGGACATGGATGAA		
TLR4-F	CTTGGTGTCGCTTTGAGTTTG	62	107
TLR4-R	GTCTCTGCTCCACTTTAGGTATG		
TLR18-F	ACAGACTAAATGGCCAGGGAAG	62	118
TLR18-R	AACCACAAGCAAGGGCAAAG		
TLR22-1-F	CCTCTTCTTAGCCTTCCTTTAC	62	94
TLR22-1-R	CTCGTCTTTGGTGTTGTAGG		
TLR22-2-F	TTCCAGGGACTGTGGTATTTG	62	98
TLR22-2-R	GCCCACAGATAAGGAGTGTAAG		
TLR22-3-F	CCATCGGCATTCTTTGGTTT	62	169
TLR22-3-R	CTGTGTTCAGGAATGCCTTG		
MyD88-F	GAGTTTCCCACTCCGTTAAGA	62	92
MyD88-R	CGCCGAGATGATGGACTTTA		
β-actin-F	GACCACCTTCAACTCCATCAT	62	126
β-actin -R	GTGATCTCCTTCTGCATCCTATC		

## Data Availability

Publicly available datasets were analyzed in this study. The rest of the data presented in this study are available on request from the corresponding author.
